# Case of Cardiac, Hepatic, and Pulmonic Disease; with the Appearances on Dissection

**Published:** 1816-04

**Authors:** J. Johnson

**Affiliations:** St. George's Square, Portsea


### *Case of Cardiac, Hepatic, and Pulmonic Disease; with the Appearances on Dissection.*

ON the 16th of February, 1816, I had an opportunity of inspecting the body of a convict, who had long lingered under a complicated complaint, attended with obscure

---

and ambiguous symptoms, of which he died in the Convict Hospital-Ship at Portsmouth, under the care of Mr. J. P. Porter, Surgeon at that Station.

No history of the disease having been particularly preserved, a bare outline of the prominent symptoms only can be here presented. "Jonathan Frost, convicted of Bigamy, was admitted into the hospital about two years ago, with symptoms of hepatic derangement; particularly fulness and tenderness in the right hypochondrium; sallowness of countenance, irregularity of bowels, &c. for which mercury was prescribed, and after a few weeks he returned to work. He did not remain well long, however, but was occasionally on the list afterwards, partly for the above-mentioned complaint, and partly for another train of symptoms which developed themselves in the thoracic region. These were cough; difficulty of lying on the *left* side; dyspnœa on any sudden exertion; and palpitatio, when agitated, or ascending a ladder.

"On the 24th of July, 1815, he was received for the last time into the hospital, and never removed from thence till he died. He had now great difficulty of breathing; total inability of lying on the left side. Morning nausea; being unable to retain any thing on his stomach in the early part of the day; pain in the *right* hypochondrium subsided, but a swelling out of the ribs on the lateral and anterior part of the *left side*, with cough, and the occasional expectoration of a caseous substance, which appeared sometimes to come from the stomach by vomiting; sometimes from the lungs by coughing. No great perturbation appeared in the vascular system. The pulse, except when using any exertion, or when he was agitated, varied from 70 to 80 strokes in a minute, regular, but wiry. The bowels were not now particularly deranged, though somewhat confined. The appetite was craving, except in the mornings, when nausea prevailed. He had *an insatiable desire for ardent spirits.* He could only sleep on his right side, and started strongly many times in the night. His mind was very desponding, and his temper exceedingly fretful; but he never exhibited any strong febrile affection, or any symptom of delirium. Till within a day or two of his death, he sat up for several hours, and smoked his pipe, of which he was very fond. In the night too, he frequently got up to smoke, his breathing being so short.

The protuberance on the left side, with a supposed obscure fluctuation, induced Mr. Porter at one time to entertain some idea of an operation for empyema; but the symptoms being anomalous and contradictory, that intention was given up. Towards the close of life, he coughed and vomited up much of the caseous substance, with some purulent matter; yet no marked symptoms of hectic fever supervened. He appeared to die from the inability to retain any food on his stomach for a sufficient length of time, for the purpose of chylification; and from the want of sleep. Anasarca of the lower extremities came on about a month before death.

"After the use of the mercury, which relieved the hepatic symptoms, no medicine appeared to afford benefit; and as there was evidently organic derangement in the thoracic viscera, no very active remedy was employed. Attention to the bowels, with anodyne and expectorant medicines, sometimes digitalis, were the only means employed."

*Appearances on Dissection, February the* 15*th*, 1816,
(Forty hours after Death.)

Body considerably emaciated; left side of the thorax opposite to the heart, and in front of that organ, projecting and very firm. On stripping back the integuments, the cartilaginous extremities of the ribs on the *left* side were ossified and entirely resisted the scalpel. They were sawed through. On the right side, the knife went freely through the cartilaginous joinings of the ribs and sternum; when a considerable quantity of yellow serum gushed out. Much difficulty was experienced in raising the sternum, so adherent was the pericardium to the inner surface. The right lung was found so diseased as to be entirely incapable of performing function. Part of it was composed of firm white tubercles, part condensed like liver, part suppurated, and the remaining space filled with water. The *left* lung was perfectly sound. The pericardium adhered firmly to, and seemed to be blended with, the surface of the heart. The *right ventricle* was greatly enlarged; and its parietes so attenuated and blended with the pericardium and pleura, that we cut into its cavity, when we thought that we were only cautiously dividing the thickened pericardium. The carneæ columnæ and chordæ tendineæ were indistinct, the former pale and flabby. The auriculo-ventricular opening, in the side of the heart, was contracted and obstructed by two cauliflower-looking excrescences that projected from opposite sides, and scarcely admitted a finger to pass between them. They occupied the space of the auriculoventricular valves. The origin of the pulmonary artery was dilated. The *left* ventricle was larger than natural, but smaller than the right. Its muscular parietes were about a third of an inch in thickness; the carneæ columnæ and chordæ tendineæ very strong and distinct. The origin and arch of the aorta were dilated to more than an inch in diameter.

*Abdomen.*—Liver scirrhous and tuberculated throughout its whole substance. The edge of the liver was nearly as hard as cartilage. The gall-bladder turgid with a black substance as viscid as birdlime. Stomach, spleen, kidnies, and intestines perfectly natural, excepting two or three approaches to stricture in the ileum, and an adhesion of the descending portion of the colon to the spleen. Pancreas scirrhous.

However deficient in minuteness of detail the history of the case may be, the data preserved are sufficient to afford some clue to the *order* of the phænomena appearing after death. The character of the patient, (a convict) his insatiate desire for alcohol during confinement, and the ostensible disease for which he was admitted into the hospital two years ago, point out the liver as the original seat of organic derangement. Its post mortem state corroborates this opinion. Although from circumstances unnecessary to mention here, I have long been in the habit of examining the biliary organ in the dead subject, I have rarely seen a more dense or scirrhous state of the organ, than in the present instance. It must have required a very considerable number of years to induce such a change.

After removing the viscus, and cutting it through in every direction, not a part of it appeared in a state capable of performing its peculiar functions. The blood-vessels themselves were in a contracted state, so that it is difficult almost to conceive how circulation, much less any secretion, could be carried on. The lung opposed to the diseased liver was destroyed. Could this be the effect of chance? Is it not strange, that tubercles should be found in the right lung, and none in the left? Must it not have been from the pressure and contiguity of a diseased liver? Independently of the manifestation of the hepatic symptoms *prior* to those of the lungs, the character of the two lesions gave a more remote date to the former than to the latter. I think it may be fairly concluded, that the *heart* was the *last* organ deranged in structure, of the three. The obstructed liver would at least molest the cardiac *functions*; instances of which we daily meet with. The pulmonic tubercles and condensed state of the right lung, must have offered a serious obstacle to the function of the pulmonic side of the heart, and may be fairly accused of producing the dilatation of the right cavity of the circulating organ. In what relation to this dilatation the diseased valves stand, whether as cause or effect, it may not be easy to decide. The dilatation of the right ventricle appeared to be a *passive* one, for its parietes were remarkably attenuated, yet inflammation cemented the pericardium to the surface of the heart. It is somewhat curious, that the original cartilages of the ribs had become ossified opposite to this diseased heart; and yet had the cardiac aneurism gone on longer, before death, these ossifications would, in all probability, have again disappeared by absorption. Did Nature take the alarm, and endeavour to protract the fatal catastrophe of passive aneurism of the heart, by ossifying the contiguous cartilages? Although the obstructed and tuberculated state of the right lung might have conduced principally to the cardiac disease, yet it is probable, that the enlargement of the heart in its turn conduced mainly to the condensation, suppuration, and effusion on that side of the thorax. This suspicion is strengthened by the fact, that the tubercular suppuration was not attended with the usual hectic febrile symptoms which accompany a natural and spontaneous suppuration of these morbid bodies in the lungs, where no cardiac lesion exists. The *decubitus difficilis* on the *left side* is worthy of notice. When, under ordinary circumstances, a tubercle is suppurating in one lung of a phthisical person, the *decubitus difficilis* is on the affected side:—Here it was just the reverse. The *sound* lung of the left side and the *diseased* heart, could not bear the pressure of a tuberculated and condensed lung when placed over them, and therefore compelled the patient to lie with the unsound lung beneath the other. The morning nausea, and the rejection of food a short time after eating are not so easily accounted for, unless by the scirrhous and enlarged pancreas on which a *distended* stomach would naturally press.

The anasarcous swellings of the lower extremities are such usual consequences of obstructed lungs, scirrhous liver, and diseased heart, that they require no remarks. The mental despondency seems to be a pretty constant attendant on cardiac diseases, as well as hepatic, and is not easily explained. As it is only by the history of symptoms during life, and the appearances on dissection, that we can judge of the *original* seat of lesion in cardi-hepatic diseases; it is to be hoped that this sketch may not be devoid of interest. Corvisart and Portal have probably strained the point too far; one is always giving the priority of organic derangement to the heart; the other, to the liver. It certainly appears more easily conceivable, however, that hepatic disease should produce functional, and ultimately structural derangement in the organ of circulation, than that an organic affection of the heart should induce a similar state of the liver. The foregoing case, and others which I have seen, tend to the same conclusion. Our practice must necessarily be modified by a discrimination, in such circumstances, of the priority of affection in one or other viscus.

A young man has just died here, who had long laboured under aneurism of the heart. For nearly two years, the action of the heart was so violent, that its pulsations could be seen from the clavicle to the umbilicus. Constant cough, with little or no expectoration, accompanied the cardiac affection. About two months ago, a dropsical effusion suddenly filled the lower extremities and scrotum to an enormous size; and such was the consequent diminution of vascular action, that all external pulsation disappeared, and the action of the heart could only be ascertained by thoracic examination. Still, however, the cough continued. This effusion appeared an effort of Nature to relieve the vascular system, and particularly its central organ, as is believed by Dr. Parry. After scarifying the legs and scrotum several times, and drawing off vast quantities of water, mortification seized both lower extremities, under which he lingered for several weeks, before he died. Leave was not obtained to open the body.

J. JOHNSON.

*St. George's Square, Portsea,*
*March* 1, 1816.

PART

